# Approaches for Reducing Expert Burden in Bayesian Network Parameterization

**DOI:** 10.3390/e27060579

**Published:** 2025-05-29

**Authors:** Bodille P. M. Blomaard, Gabriela F. Nane, Anca M. Hanea

**Affiliations:** 1 Department of Applied Mathematics, Delft University of Technology, 2628 CD Delft, The Netherlands; bodille.blomaard@outlook.com; 2 Centre of Excellence for Biosecurity Risk Analysis, University of Melbourne, Melbourne, VIC 3010, Australia; anca.hanea@unimelb.edu.au

**Keywords:** Bayesian networks, expert judgment, InterBeta, RNM, functional interpolation

## Abstract

Bayesian networks (BNs) are popular models that represent complex relationships among variables. In the discrete case, these relationships can be quantified by conditional probability tables (CPTs). CPTs can be derived from data, but if data are not sufficient, experts can be involved to assess the probabilities in the CPTs through Structured Expert Judgment (SEJ). This is often a burdensome task due to the large number of probabilities that need to be assessed and the structured protocols that need to be followed. To lighten the elicitation burden, several methods have previously been developed to construct CPTs using a limited number of input parameters, such as InterBeta, the Ranked Nodes Method (RNM), and Functional Interpolation. In this study, the burden/accuracy trade-off of InterBeta is researched by applying the method to reconstruct previously elicited CPTs and simulated CPTs, first by comparing these CPTs to ones constructed using RNM and Functional Interpolation. After that, InterBeta extensions are proposed and tested, including an extra mean function (shifted geometric mean), the elicitation of additional middle rows, and the newly proposed extension ExtraBeta. InterBeta with parent weights is found to be the best-performing method, and the ExtraBeta extension is found to be promising and is proposed for further exploration.

## 1. Introduction

Bayesian networks (BNs) are powerful probabilistic graphical models widely used to represent complex systems in fields such as healthcare [1] and nuclear safety [2]. A BN is defined by an acyclic graph, whose nodes represent random variables and directed arcs represent potential dependencies between the variables (more precisely, the lack of arcs represents (un)conditional independence statements). Arcs are directed from parent node(s) to child node(s), suggesting that a child is dependent on or caused by a parent. Both discrete and continuous distributions can be used to model the variables represented by the nodes. For each node, a (conditional) probability distribution can be specified. When all variables are discrete, the (conditional) probabilities are summarized in conditional probability tables (CPTs).

CPTs can be estimated from data, and it is noteworthy that their size grows exponentially with the number of parent nodes, making large discrete BNs with many nodes and variable states computationally intensive. In cases where certain parts of the data are not available, various imputation methods have been developed based on BNs (see, for example, [3,4,5]).

With sparse or completely missing data, CPTs must be estimated through expert elicitation. Structured Expert Judgment (SEJ) methods, including the Delphi method [6], the Classical Model [7], and the IDEA protocol [8], facilitate this process by systematically gathering expert input while managing uncertainty. The Delphi method collects individual expert assessments over multiple rounds, allowing updates based on shared information, and aggregates results using equal weights. The Classical Model improves this by weighting experts’ uncertainty assessments based on their performance on calibration questions, reflecting the statistical accuracy (also known as calibration) and informativeness of their uncertainty assessments. Calibration questions are questions whose answers are known to the analyst but not to the experts at the moment of the elicitation. The IDEA protocol combines initial individual assessments with a discussion phase, followed by final assessments and aggregation, using either equal or performance-based weights.

As mentioned previously, CPTs can become very large for complex BNs, making full expert elicitation resource-intensive. To ease this burden, methods like Noisy-OR [9], the Ranked Nodes Method (RNM) [10], InterBeta [11], and Functional Interpolation [12] reduce the number of required probability inputs. However, their application and comparative performance remain underexplored. Although some methods have been tested individually or against data [13,14,15], a comprehensive comparison is lacking.

This research evaluates methods for parameterizing BNs and their CPTs in situations where data are unavailable, thus requiring expert elicitation. By assessing the limitations of existing methods (as mentioned above) and comparing results from expert-elicited and simulated CPTs, this study aims to identify approaches that best balance accuracy and expert effort, ultimately providing practical guidance for method selection in data-sparse environments.

### 1.1. Related Works

We begin with a recap of the existing methods and approaches found in the literature, their applications, existing comparisons, and most notable findings.

The Noisy-OR method, RNM, InterBeta, and Functional Interpolation have all been applied to varying extents, with Noisy-OR being the most studied due to its longer history.

The applicability of the Noisy-OR(/MAX) method has been studied previously on existing BNs. It was found that the Noisy-MAX gate provides a good fit for as many as 50% of CPTs in two out of three BNs [16]. Another study investigated the effect of using a leaky Noisy-OR version of a BN, which was used for the early detection of classical swine fever in pigs, reducing the number of necessary parameters from 470 to 348. It was cautiously concluded that the method can indeed be applied for diagnostic applications. More recently, applications of the Noisy-OR/MAX method include the development of BNs for the investment strategies of farmers [17], the occurrence of hydrogen leakage in proton-exchange membrane fuel cells [18], and construction project risk [19]. The reduction in the elicitation burden is logarithmic, meaning that the Noisy-OR(/MAX) method requires parameters on the order of the number of parents rather than the full CPT.

Other methods include the EBBN method [20], Cain’s method [21], and Røed’s method [22]. A comparative study investigated the performance of the RNM, Functional Interpolation, the EBBN method, Cain’s method, and Røed’s method [14]. The findings highlighted the need to address strong factor influences and uncertainty. While Functional Interpolation performed best in this regard, it imposed a higher elicitation burden. The study also noted challenges in representing multi-factor influences for the EBBN method, Cain’s method, and Røed’s method.

RNM has been used to model teamwork quality in agile teams [23] and, alongside Noisy-OR, for disaster assessment in the oil and gas supply chain [24]. It has also been combined with other methods (e.g., DEMATEL, which stands for Decision-Making Trial and Evaluation Laboratory and is a popular causal modeling approach in the multiple criteria decision-making domain) to construct BNs in a case study with 14 experts from the automobile industry [25]. Functional Interpolation has been applied in dynamic BN modeling to assess the residual life of corroded subsea pipelines [26], with suggestions for combining it with actual data to reduce expert burden.

InterBeta has not yet been used to reconstruct full CPTs, although it has been tested on fully elicited BNs [27]. One of the InterBeta versions produced CPTs similar to fully elicited ones, but its performance with fully elicited parameters remains untested.

We selected methods for further investigation based on two factors: (1) the promising accuracy and efficiency in parameter reduction identified in the literature review, and (2) the methods’ potential for operationalizing parameters, making them easier for experts to assess.

### 1.2. What to Expect in This Paper

This study examines the trade-off between the elicitation burden and the accuracy of CPT reconstruction by first comparing InterBeta with RNM and Functional Interpolation. Extensions of InterBeta are proposed and compared with the InterBeta method. The next section describes these extensions, along with proposed extensions for RNM and Functional Interpolation.

Section 2 commences with an overview of the data used to test the construction methods. The term “data” in this context means “expert-elicited data” rather than observed/measured data. The section details the InterBeta method, including its variations and extensions; provides brief overviews of the RNM and Functional Interpolation methods; and concludes with a summary of the measures used to assess the accuracy and elicitation burden of each method.

Section 3 presents the results, starting with a comparison of InterBeta, RNM, and Functional Interpolation. It then focuses on InterBeta and its extensions, concluding with a discussion on the trade-off between the elicitation burden and accuracy, and offering guidance on best practices for applying InterBeta.

## 2. Methodology

The Functional Interpolation, RNM, and InterBeta CPT construction methods were implemented in Python, along with algorithms to find the optimal input parameters for each method. All analyses were conducted on a laptop running Windows 11. The system was equipped with an Intel Core i7 and 16GB of RAM. Computations were performed using Python v3.11.5. The optimization algorithms aim to find the optimal input parameters by minimizing the difference between the “true” CPT and the reconstructed CPT. The code for each of the methods, including the optimization algorithms and the generation of simulation data, was written by one of the co-authors (Bodille Blomaard) and is available at https://github.com/Bodille1/BN_parameterization, accessed on 28 May 2025. The difference measures used to optimize these parameters are later also used to determine the reconstruction accuracy and are presented at the end of this section. In addition to the reconstructed CPTs’ accuracy, the performance of a CPT construction method also considers the elicitation burden. The elicitation burden is measured by the number of parameters, as no alternative burden metric exists for different parameter types.

This section provides an overview of the data—both previously elicited and simulated CPTs—followed by an introduction to InterBeta, its variations, and proposed extensions. Then, RNM and Functional Interpolation, along with their respective extensions, are presented. For a more detailed presentation of these methods, see [28].

### 2.1. Data

The term “data” in this context means elicited data; more precisely, elicited entries/parameters of the BN-associated CPTs. Moreover, these elicited CPTs need to correspond to ordinal discrete variables, whose dependencies are represented by a BN whose nodes are ranked, and the states of a ranked node are ordered in terms of their influence on the child node. The InterBeta, RNM, and Functional Interpolation approaches all assume that the highest (lowest)-ranked state of the parent node shifts the mean of the child node’s distribution toward its highest (lowest)-ranked state the most.

The availability of such fully expert-elicited CPTs for BNs is limited, and among those available, the data are often not publicly accessible. A noteworthy exception is BayesFusion’s public model repository https://www.bayesfusion.com/bayesbox/, accessed on 16 October 2024.

This study uses fully elicited CPTs from three recent BN studies, as well as simulated CPTs. The elicited data are introduced first in the context of separate BNs. Afterward, the simulation method used to generate the CPTs is briefly outlined. Both the elicited and simulated full CPTs are treated as the “ground truth” for evaluating the accuracy of the partially specified CPTs (corresponding to the different selected approaches).

#### 2.1.1. Elicited Data

Data were collected from three distinct BNs, yielding 34 unique CPTs across the following applications: the abundance of pollinators (bees) in the UK [29]; household food security in Victoria, Australia [30]; and the persistence of polar bears [31].

##### Pollinator Abundance (PA)

**Figure A1:**
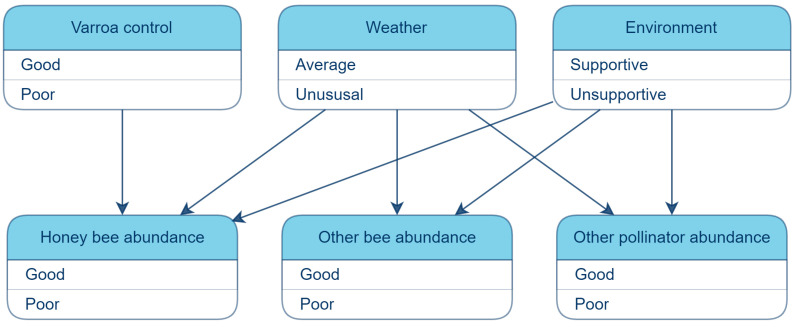
Bayesian network structure of the pollinator abundance study.

**Figure A2:**
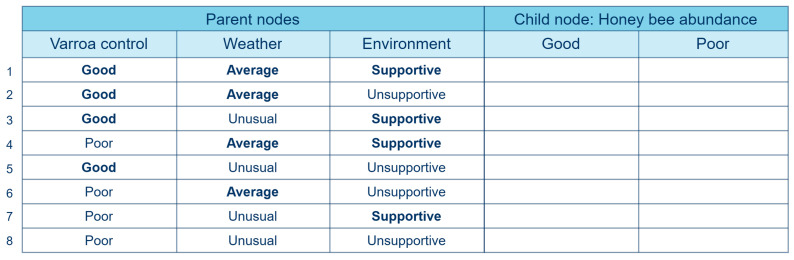
Unelicited conditional probability table for the honey bee abundance child node in the pollinator abundance Bayesian network.

The first BN application used in this paper models the abundance of pollinators in the UK [29]. Since disease pressure, weather, and the environment affect pollinator abundance, they are modeled as its parents in the BN. The full BN is shown in Figure A1 in Appendix A, along with the CPT used here in Figure A2.

All CPTs were fully elicited using the IDEA protocol [8] from a group of ten experts. Thirty-two CPT values were elicited, including 16 for bee abundance and 8 for each of the other child nodes. The experts provided best estimates and credible intervals for each value over two elicitation rounds. Initially, the parent nodes were intended to have more than two states, but this number was reduced to ensure that the elicitation could be completed in time.

The experts also participated in a calibration exercise, which revealed no significant differences in the calibration scores. The BN was ultimately parameterized using an equally weighted combination of expert assessments. In the context of the Classical Model for SEJ, the final aggregation of judgments is called a decision maker (to indicate that a decision maker adopts the aggregated group judgment as their own). The equally weighted combination of expert assessments is called an equal weight decision maker (EWDM).

For this study, only the CPT for the ‘honey bee abundance’ node was considered. All other CPTs were small enough to allow full elicitation. The comparison dataset included individual expert assessments, along with the EWDM and the performance-based weight decision maker (PWDM).

##### Food Security (FS)

The next BN application focuses on household food security in Victoria, Australia [30]. The BN models the influence of physical access, availability, and equivalized income on food security levels. This BN is part of a broader, integrated decision support system for food security [32].

Similar to the pollinator abundance study, the IDEA protocol was used to elicit all parameters. Five experts participated, and a total of 48 values were elicited to complete the CPT, along with additional calibration questions. The final elicited values were aggregated using performance-based weights. The study revealed that one parent node had a dominant effect on the child node. While the effects of physical access and food availability on food security levels were significant, household equivalized income was by far the strongest determinant. As above, individual assessments, the EWDM, and the PWDM were used for comparison.

##### Polar Bears (PB)

The final BN considered in this study examines the relative influence of environmental and anthropogenic stressors on polar bear persistence [31]. This BN is significantly larger than the previous examples and comprises 48 nodes. Among these, the CPTs of 21 nodes were elicited from experts, while the remaining 27 nodes are root nodes defined by uniform marginal distributions. The CPTs were constructed through a consensus process, in which an Excel file was circulated among a team of eight experts. Unlike the previous examples, the elicitation did not use one of the SEJ methods discussed in Section 1, and no calibration exercise was conducted. As a result, each CPT had only a single final assessment.

In this study, we use a subset of 16 unique CPTs.

#### 2.1.2. Simulated Data

While the three elicited BNs and their CPTs are valuable, they are insufficient for generalizing the results. Ideally, more elicited BNs with diverse dependence structures would be analyzed. In their absence, simulated data—reflecting similar and varied dependence patterns—serve as the next best alternative. It is noteworthy that the simulated data are not aimed at replicating how experts provide assessments, but rather at obtaining full CPTs to enable the analysis of the considered methods.

Even though this study concerns discrete BNs and dependencies represented as CPTs, there is a strong theoretical and practical relationship between rank correlation structures for discrete ordinal variables and their continuous counterparts (ordinal discrete random variables can be written as monotone transformations of uniform variates). We use this relationship, as defined in [33], to justify the use of Spearman’s rank correlation matrices in the simulations to both represent the dependence in the elicited CPTs and to simulate different dependence structures.

A BN with four nodes (one child node and three independent parent nodes) is considered. Spearman’s rank correlation matrices are calculated from samples drawn from the CPTs (for details on the simulation exercise, see [28], and for details on the theoretical underpinning of using rank correlations to approximate the dependence between ordinal discrete variables, see [34]). Four types of correlation structures are chosen between the child node and the parent nodes based on the observed correlation structures in the PA, FS, and PB studies:

- Equal and low (EqL): The same low correlation (0.23) is set between each parent node and the child node.
- Equal and high (EqH): The same high(er) correlation (0.37) is set between each parent node and the child node.
- Increasing (Incr): The correlations between the parent nodes and the child node increases, with values of 0.15, 0.30, and 0.45 for the three parent–child pairs, respectively.
- Outlier (Out): The correlation between one parent node and the child node is significantly higher than the correlation between the other parent nodes and the child node, with values of 0.1, 0.1, and 0.9 for the three parent nodes, respectively.

These matrices were used to sample data on which a discrete BN can be fitted. The full details of the method are presented in [28].

### 2.2. InterBeta

InterBeta [11] is a method for constructing CPTs through interpolation when only best- and worst-case scenarios are available. InterBeta assumes ranked nodes with weighted parental influence, which are combined using an independent function. The core assumption of the method is that the distribution of the child node can be approximated by a beta distribution. Note that the beta distribution can take on a uniform (α,β=1), unimodal (α,β>1), or bimodal (0<α,β<1) shape within the interval [0,1]. This section describes the InterBeta method, its existing variants, and proposed extensions.

#### 2.2.1. Method

Given a CPT structure and an empty table with assigned parent node state combinations for each row, CPT values are calculated by interpolating the parameters of the beta distribution between the best and worst rows. Note that a CPT row refers to the conditional distribution of the child node for a certain combination of parent node states. For example, the best row is a multinomial that describes the distribution of the child node when all of the parent nodes are in their most positive state.

To start, experts provide the best and worst rows of the CPT by assessing the multinomial distributions or by providing beta distribution parameters, either by specifying the α and β parameters directly or by providing the mean and standard deviation. If multinomials are provided, InterBeta fits the beta distributions for the best and worst rows using the method of moments and the following optimization strategy:

1. The mean (μ) and variance ($\sigma^{2}$) are derived from the multinomial distribution, and the alpha (α) and beta (β) parameters are then calculated using the method of moments [11].
2. The α and β parameters are iteratively adjusted using Gaussian noise, accepting only those mutations that improve the Kullback–Leibler (KL) divergence between the discretized fitted beta distribution and the original elicited multinomial distribution. This process typically converges within 1000 iterations [11].

The α and β parameters of the intermediate rows between the best and worst rows are then derived through interpolation. To calculate these parameters, each CPT row is assigned a weight $W_{k}\in \left[0,1\right]$, where the best and worst rows have weights of 1 and 0, respectively. If the worst row is approximated by $Beta(\alpha^{↓},\beta^{↓})$, and the best row by $Beta(\alpha^{↑},\beta^{↑})$, then the parameters can be calculated using the following equations:

(1) $$\begin{array}{c} \begin{array}{cc} {\hat{\alpha }}_{k}=g_{\alpha }\left(x_{k}\right) & =W_{k}\cdot \alpha^{↑}+\left(1-W_{k}\right)\cdot \alpha^{↓},\\ {\hat{\beta }}_{k}=g_{\beta }\left(x_{k}\right) & =W_{k}\cdot \beta^{↑}+\left(1-W_{k}\right)\cdot \beta^{↓}. \end{array} \end{array}$$

The row weight ($W_{k}$) can be either elicited directly from experts or calculated based on expert-elicited parent weights ($w_{i}$) and state weights ($\omega_{i,j}^{k}$). In this case, the modeler can decide to aggregate the weights using one of the following mean functions:

$$\begin{array}{ccc} \mathrm{arithmetic}\;\mathrm{mean}:\;\; & W_{k}=\frac{1}{n}\sum_{i=1}^{n}\omega_{i,j}^{k},\;\;\;\;\;\;\;\mathrm{geometric}\;\mathrm{mean}:\;\; & W_{k}={\left(\prod_{i=1}^{n}\omega_{i,j}^{k}\right)}^{\frac{1}{n}},\\ \mathrm{shifted}\;\mathrm{geometric}\;\mathrm{mean}:\;\; & W_{k}={\left(\prod_{i=1}^{n}\left(\omega_{i,j}^{k}+\delta \right)\right)}^{\frac{1}{n}}-\delta ,\;\;\mathrm{harmonic}\;\mathrm{mean}:\;\; & W_{k}=\frac{n}{\sum_{i=1}^{n}\frac{1}{\omega_{i,j}^{k}}}, \end{array}$$

where *k* is the row index. Note that the parent state weights depend on the parent weights in the following way: $\;\omega_{i,1}=w_{i},\;\;\omega_{i,2}=w_{i}-\frac{w_{i}}{s_{C}-1},\;\;\omega_{i,3}=w_{i}-\frac{2w_{i}}{s_{C}-1},\;\;\ldots ,\;\;\omega_{i,s_{C}}=0$, for parent node *i* in state *j*, and for $s_{C}$ the number of child states. The parent and state weights can be elicited from experts by considering, for example, weights proportional to the correlations between the given nodes. To the best of our knowledge, no structured protocol exists for eliciting those weights. Alternatively, default values can be used, i.e., $w_{i}=1$ for all $i=1,\ldots ,s_{C}$.

The arithmetic and geometric means were employed in the pollinator abundance and household food security CPTs [27], and the harmonic mean was also suggested as an option. However, both the harmonic and geometric means have a major drawback: they yield zero if any entry is zero. Given that the weight of a node’s worst state is zero, these mean functions result in a zero weight for all rows where at least one parent node is in its worst state. To address this issue, a straightforward extension is proposed: the “shifted geometric mean”. A constant (δ>0) is added to each entry in the geometric mean and then subtracted from the result. This avoids the issue of zero entries affecting the mean calculation. In the analysis of the (simulated) data, the constant δ was set to one, based on trial and error.

Finally, the CPT values are derived by discretizing the beta distributions found. The [0,1] interval is divided into $s_{C}$ equally spaced intervals, corresponding to the number of child states. The multinomial distribution is then derived by calculating the probability mass for each interval. For a combination of parent states $X_{k}=x_{k}=\left(X_{1}=x_{1}^{k},\ldots ,X_{n}^{k}=x_{n}\right)$, the child node distribution obeys the following conditional probability:

(2) $$\left(X_{C}\;\;|\;\;X_{k}\right)∼Beta\left(g_{\alpha }\left(x_{k}\right),g_{\beta }\left(x_{k}\right)\right),$$

where *k* indicates a particular combination of parent states and the functions $g_{\alpha }$ and $g_{\beta }$ are the interpolated distribution parameters based on the relationship in Equation (2). The cumulative distribution function (cdf) of the beta distribution, denoted by $F(x;{\hat{\alpha }}_{k},{\hat{\beta }}_{k})$, is discretized to calculate the CPT values

(3) $$\left(X_{C}=x_{C}^{i}\;|\;X_{1}=x_{1}^{k},\ldots ,X_{n}=x_{n}^{k}\right)=F\left(\frac{s_{C}+1-i}{s_{C}};{\hat{\alpha }}_{k},{\hat{\beta }}_{k}\right)-F\left(\frac{s_{C}-i}{s_{C}};{\hat{\alpha }}_{k},{\hat{\beta }}_{k}\right),$$

for $i=1,\ldots ,s_{C}$, where $s_{C}$ represents the number of states of the child node.

#### 2.2.2. Versions

The InterBeta method has several versions: best–worst rows, parent weights, parent state weights, and row weights, depending on which input parameters are elicited from experts. Table 1 gives an overview of the parameters that need to be elicited for each version of the InterBeta method. For each version, the best and worst CPT rows need to be elicited. The best and worst rows represent scenarios where all parent nodes are in their most positive state and their least positive state, respectively.

#### 2.2.3. Extensions

This study proposes several extensions of the InterBeta method. The first extension involves aggregating parent weights using a shifted geometric mean. A key limitation of the InterBeta method lies in its rigid requirement for experts to assess only the best and worst rows of the CPT. This inflexibility can be restrictive in certain contexts. To address this limitation, two significant extensions are introduced in this study: the inclusion of an additional intermediate elicited row between the best and worst rows, and the option for experts to assess “good” and “bad” rows instead.

The former involves eliciting not only the best and worst rows but also one or more intermediate rows. This approach increases the flexibility of the beta parameters and potentially improves the performance of InterBeta. Specifically, an intermediate row where all parent nodes are in states between their best and worst states (referred to as middle states) is considered. This choice balances the extremes while avoiding the expert burden that would arise from eliciting many extreme rows, as seen in methods like Functional Interpolation [12].

The latter proposed extension, ExtraBeta, diverges only in interpolation by allowing experts to assess scenarios within their frame of knowledge. Similar to the weighted-sum algorithm [35], experts group compatible parent configurations. Here, instead of the best and worst rows, experts select a “good” row and a “bad” row themselves for elicitation, which are part of the upper and lower halves of the CPT, respectively. This entails that the default weight of a good row is greater than 0.5 and that the default weight of a bad row is less than 0.5. In addition, for each combination of parent states, the mean value of the elicited multinomial of the good row must be strictly greater than the mean of the elicited multinomial of the bad row. The remaining CPT values are then constructed via interpolation and extrapolation of the beta parameters. It is noteworthy that ExtraBeta retains InterBeta’s flexibility in weight options, including parent weights, parent state weights, and row weights. Notably, the elicitation burden is increased by two parameters in comparison to InterBeta since experts are asked to select two scenarios for elicitation. For the InterBeta method, the scenarios are fixed.

A potential limitation of ExtraBeta is that if experts do not assess the same CPT rows, their assessments cannot be aggregated before constructing the full CPT. However, prior research suggests that the difference between interpolating aggregated assessments and aggregating interpolated assessments is insignificant [27].

### 2.3. Other CPT Construction Methods

For comparison purposes, the performance of the InterBeta method is first assessed against the Ranked Nodes Method (RNM) and the Functional Interpolation method. These two methods are not discussed in detail here; only their most important characteristics are reviewed, and some extensions are introduced. Reference [28] provides a detailed overview of these methods.

#### 2.3.1. Ranked Nodes Method

The Ranked Nodes Method (RNM) [10] is frequently used with discretized continuous nodes. The method requires a weight function to be selected by the analyst, as well as a set of weights and a variance parameter for the child node distribution. Experts are encouraged to identify appropriate parameters in multiple stages using trial and error. The CPT is then calculated by assuming that a truncated normal distribution describes the child node distribution. For a more detailed description, see [28].

One of the main flaws of RNM is its reliance on experts to use trial and error to determine the weight and variance parameters. Therefore, an extension (AutoRNM) has been developed [28] and is proposed in this study to automate the process. AutoRNM incorporates an automated parameter determination for RNM. That is, instead of eliciting a weight function, a set of weights, and a variance parameter from experts, a total of 2(n+1) CPT rows are elicited, where *n* is the number of parent nodes. The rows that need to be elicited include the best and worst CPT rows, and extra rows are chosen such that all parent nodes are in their best state, except for one that is in its worst state, or vice versa. The necessary parameters for RNM are then found by optimizing the output of RNM to fit the elicited CPT rows.

#### 2.3.2. Functional Interpolation

Similar to InterBeta, the Functional Interpolation method relies on interpolating probability distribution parameters for constructing CPTs [12]. Experts are required to assess a total of $2^{n}$ CPT rows, where *n* is the number of parent nodes, one for each combination of the best and worst states of the parent nodes. The method originally assumed the normal distribution for describing the child node distribution. To extend the method, the beta distribution and truncated normal distributions are included in this study as possible child node distributions.

### 2.4. Performance Metrics

To assess the performance of a CPT construction method, several measures can be used that reflect the accuracy and the elicitation burden of a construction method. It is assumed that the originally elicited CPTs, or the simulated CPTs, are the true CPTs. The accuracy measures used in this paper are the mean Kullback–Leibler (KL) divergence and the percentage of agreement. The elicitation burden is measured by the number of parameters that experts are asked to assess.

#### 2.4.1. Kullback–Leibler Divergence

The KL divergence of *P* from *Q* can be interpreted as the expected surprise when using *Q* as a model when the actual distribution is *P*. A KL divergence close to zero shows that the two distributions are very similar. If both *P* and *Q* are probability mass functions (pmfs) that have *s* possible states, the metric is defined as

(4) $$D_{\mathrm{KL}}\left(P∥Q\right)=-\sum_{i=1}^{s}P\left(i\right)\;\log\frac{Q(i)}{P(i)}.$$

Applied to CPTs, the KL divergence is measured between each CPT row, which can then be averaged into a single measure. It would also be possible to compare the KL divergence between the joint distributions, but this also depends on the KL divergence of the conditional distributions, as shown in [28]. For a combination of parent node states $k:(x_{1}^{k},\ldots ,x_{n}^{k})$, the KL divergence is calculated as

$$\begin{array}{c} D_{KL}^{k}\left(P\left(X_{C}\;|\;X_{1},\ldots ,X_{n}\right)∥Q\left(X_{C}\;|\;X_{1},\ldots ,X_{n}\right)\right)=\sum_{x_{C}\in X_{C}}P\left(x_{C}\;|\;x_{1}^{k},\ldots ,x_{n}^{k}\right)\ln\frac{P(x_{C}\;|\;x_{1}^{k},\ldots ,x_{n}^{k})}{Q(x_{C}\;|\;x_{1}^{k},\ldots ,x_{n}^{k})}, \end{array}$$

where, for ease of notation, we replaced $X_{i}=x_{i}$ with $x_{i}$, for i=1,…,n,C. The mean KL divergence between two CPTs is defined as

$$\begin{array}{c} D_{KL}\left({\mathrm{CPT}}_{P}\;∥\;{\mathrm{CPT}}_{Q}\right)=\frac{1}{\#\mathrm{CPT}\;\mathrm{rows}}\sum_{k=1}^{\#\mathrm{CPT}\;\mathrm{rows}}D_{KL}^{k}\left(P\left(X_{C}\;|\;X_{1},\ldots ,X_{n}\right)∥Q\left(X_{C}\;|\;X_{1},\ldots ,X_{n}\right)\right). \end{array}$$

We note that the mean KL divergence aims to capture good overall performance, or its lack thereof, while it may fail to detect significant differences (or agreement) for single conditional probabilities.

#### 2.4.2. Percentage of Agreement

A more intuitive measure is the percentage of agreement on the most likely state the child node can take in different scenarios. This measure was used for testing the performance of the EBBN method [20]. For each CPT row, it is checked whether the two CPTs “agree” on which child node state has the largest probability.

For $S=\prod_{i=1}^{n}s_{i}$ combinations of parent node states, it is checked whether the corresponding child states are equal for the constructed CPT and the true CPT:

$$\mathrm{Agreement}\;\%=\frac{100}{S}\sum_{k=1}^{S}\delta \left\{x_{C}^{k}={\hat{x}}_{C}^{k}\right\}\;,$$

where $x_{C}^{k}$ is the child state with the highest probability for the combination of parent states *k* in the true CPT and ${\hat{x}}_{C}^{k}$ is the child state with the highest probability for the combination of parent states *k* for the constructed CPT. Thus, $\delta \{x_{C}^{k}={\hat{x}}_{C}^{k}\}=1$ when the true and constructed CPTs give the highest probability to the same child node state. So, for this performance measure, a value close to 100% is desired.

#### 2.4.3. Burden

The process of eliciting parameters from experts imposes a burden that can be quantified by the number of parameters to be elicited. As the complexity of a BN increases, so does the number of parameters, leading to a greater burden on experts. For a node with $n_{s}$ states, for which parent *i*, where i=1,…,k, has $n_{i}$ states, the total number of parameters gives the $CPT\;size=n_{s}\cdot \prod_{i=1,\ldots ,k}n_{i}$. So, the CPT of a node with three parent nodes, where each node has three states, requires the elicitation of 81 conditional probabilities. Adding another parent node with three states increases the elicitation requirement to 243 probabilities. This illustrates the exponential growth in the number of parameters with the addition of nodes in a BN. Increasing the number of states of the nodes leads to a similar increase in the elicitation burden.

However, not all parameters are equally complex to assess for experts. For example, when dealing with a child node that has two states, only one probability needs to be directly assessed, since the second can be deduced, which reduces the overall burden. Along with probabilities, other parameters such as weights, variance, or correlation structures can also be elicited. While probabilities might be quite intuitive to assess, other parameters may be less “natural” to assess. Unfortunately, quantifying these subtle differences is very difficult, if not impossible.

In this work, the elicitation burden of a CPT construction method is quantified by the number of parameters required to be elicited. Although different types of parameters may impose varying levels of elicitation burden, this study assumes that the burden is equivalent across parameter types due to the lack of a more precise metric.

## 3. Results

This section presents the results, starting with a comparison of InterBeta, RNM, and Functional Interpolation in terms of elicitation burden and CPT reconstruction accuracy, including InterBeta’s performance with elicited middle rows. It also examines the results for the shifted geometric mean and ExtraBeta.

### 3.1. Comparison

To compare the performance of the InterBeta, RNM, and Functional Interpolation methods, the elicited or aggregated data from the pollinator abundance, food security, and polar bear BNs were used. The comparisons included the AutoRNM extension and the extension of InterBeta, where middle rows were also elicited. For the Functional Interpolation method, the results are shown for the best-fitting probability distribution, and for InterBeta, the results are shown for the best combination of the interpolation parameters and mean function. These parameters were optimized such that the constructed CPTs fitted as closely as possible to the true CPTs by minimizing the mean KL divergence. For more details, see [28].

**Figure 1:**
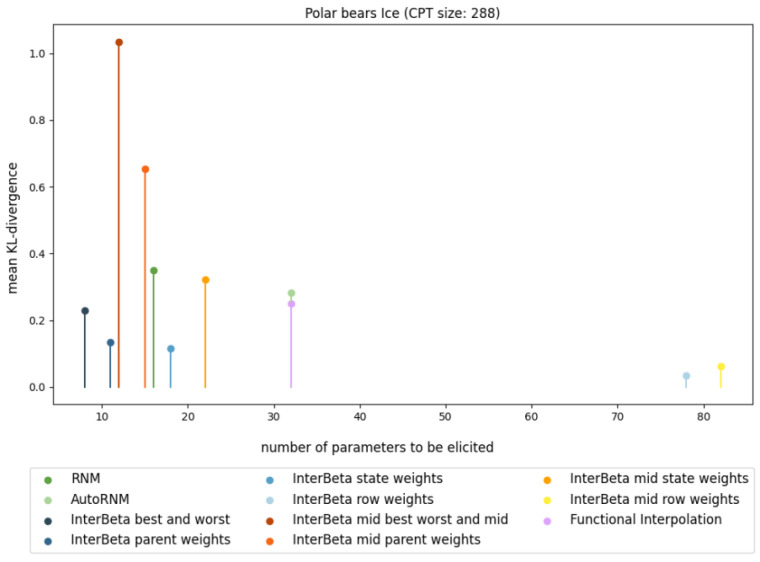
Comparison of mean KL divergence results with respect to the number of elicited parameters for PB Ice CPTs constructed using variants of the InterBeta (blue, red, and orange), RNM (green), and Functional Interpolation (purple) methods.

**Figure A3:**
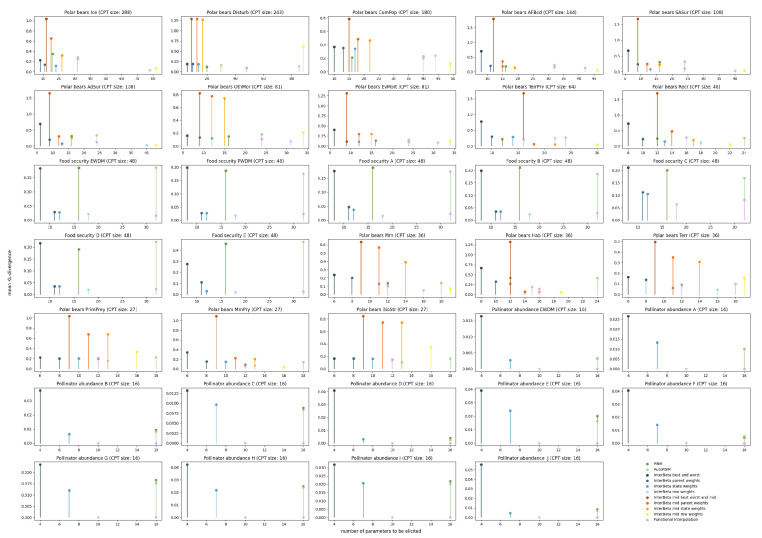
Comparison of the KL divergence between elicited CPTs and constructed CPTs for the following methods: variants of the InterBeta (blue, red, and orange), RNM (green), and Functional Interpolation (purple).

The results of the comparison between the CPT construction methods for one fully elicited CPT are presented in Figure 1 and Table 2, where performance is measured in terms of the KL divergence, and the burden is measured in terms of the number of parameters that need to be elicited. The PB Ice CPT was chosen because it was the largest CPT included in the elicited data and therefore shows the differences in performance and elicitation burden clearly. A full overview of the comparison of the CPT construction methods, including all elicited CPTs, can be found in Figure A3 and Table A1, Table A2, Table A3 in Appendix A.

The Ice CPT shows that the InterBeta method with row weights achieved the lowest mean KL divergence; nonetheless, it required the elicitation of 78 parameters. The original InterBeta method, using only the best and worst rows, outperformed RNM, AutoRNM, and Functional Interpolation while requiring fewer parameters than these methods. Introducing parent weights reduced the KL divergence by nearly 30%, although further eliciting parent state weights provided only a marginal additional improvement of about 8%. Thus, for this CPT, InterBeta with parent weights offered an efficient balance, achieving a significant reduction in the number of parameters (96%) to be elicited compared to full elicitation.

In summary, InterBeta, particularly when combined with parent weights, offers an effective approach to CPT construction with reduced parameter elicitation while maintaining competitive accuracy. It should also be noted that the accuracies of RNM and AutoRNM were similar throughout the comparison, and only the number of parameters that needed to be elicited differed. This would suggest that RNM outperformed AutoRNM, but it does not consider the relative burden of eliciting different parameters.

### 3.2. InterBeta Extensions

Three extensions of InterBeta are introduced in this paper: the elicitation of middle rows, the addition of a new mean function (shifted geometric mean), and ExtraBeta. The results of InterBeta with elicited middle rows have already been discussed in Section 3.1; therefore, they are not included in this section.

#### 3.2.1. Shifted Geometric Mean

**Figure 2:**
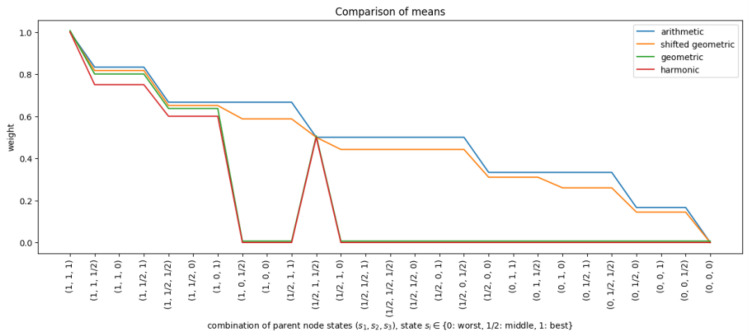
Row weights when the arithmetic mean (blue), geometric mean (green), shifted geometric mean (orange), or harmonic mean (red) was used for a node with three parent nodes, each with three states $\left(x_{1},x_{2},x_{3}\right)$, where $x_{i}\in \left\{0,\frac{1}{2},1\right\}$, and the parent and state weights were set to their default values.

The shifted geometric mean was evaluated alongside the arithmetic, geometric, and harmonic means. First, all means were used to calculate row weights for a given BN with three parent nodes and one child node. Figure 2 shows the row weights calculated by the different functions for all CPT rows, ordered from best to worst. The x-axis represents the CPT rows $(x_{1},x_{2},$$x_{3})$, where $x_{i}\in \left\{0,\frac{1}{2},1\right\}$ denotes the state of parent *i*. The figure highlights that the calculated row weights were zero for a large portion of the CPT when either the geometric mean or harmonic mean was used. This is because these means return zero when at least one of the input weights is zero. The shifted geometric mean behaved like a slightly smoothed version of the arithmetic mean.

**Figure 3:**
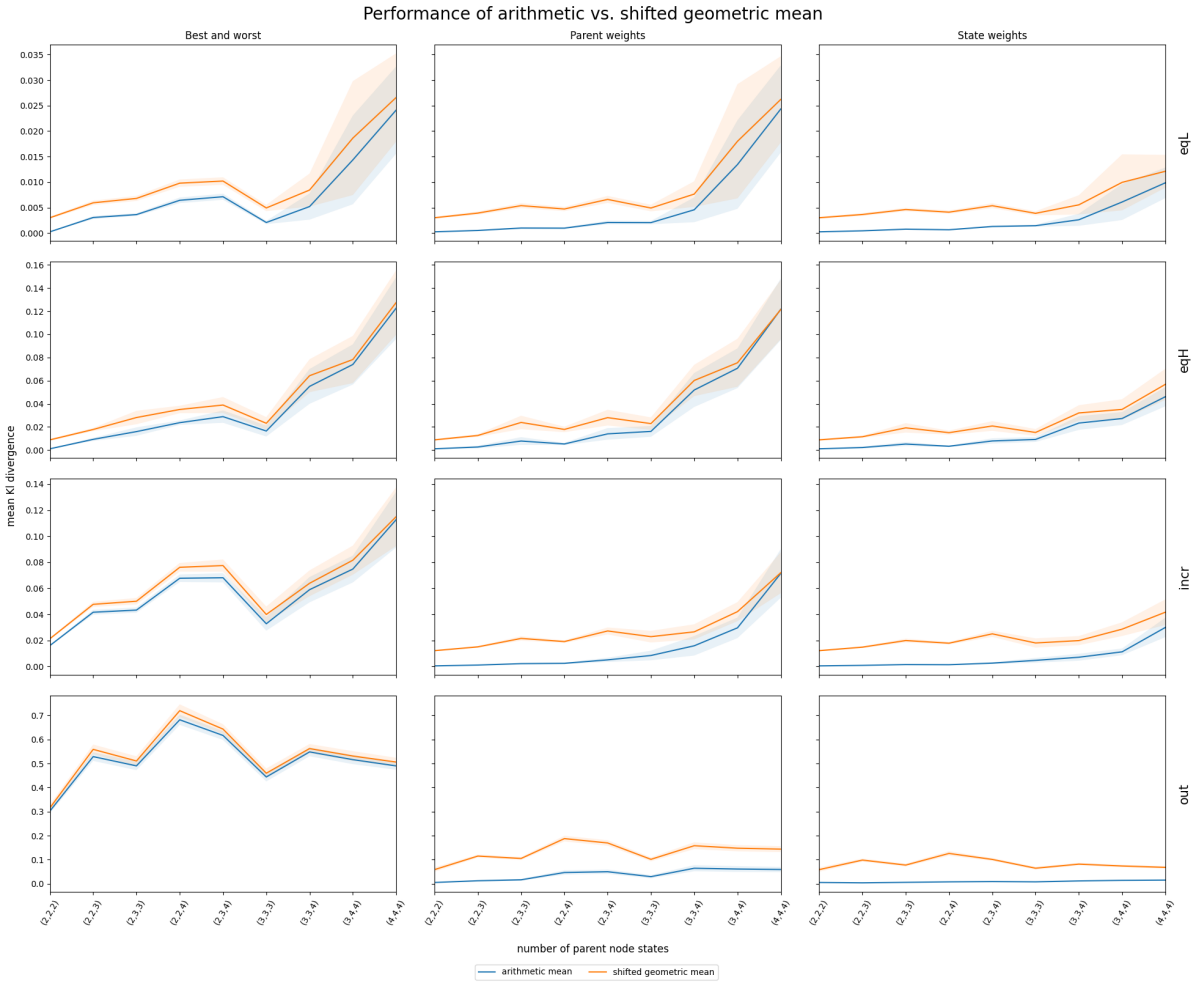
Mean and 95% confidence interval of InterBeta performance (KL divergence) over 100 replications using simulated data with four different correlation structures (EqL, EqH, Incr, and Out) when the arithmetic (blue) and shifted geometric (orange) means were considered, with α and β as interpolation parameters. The CPT size increases on the x-axis scale $\left(s_{1},s_{2},s_{3}\right)$, where $s_{i}$ is the number of states for parent *i*.

**Figure A4:**
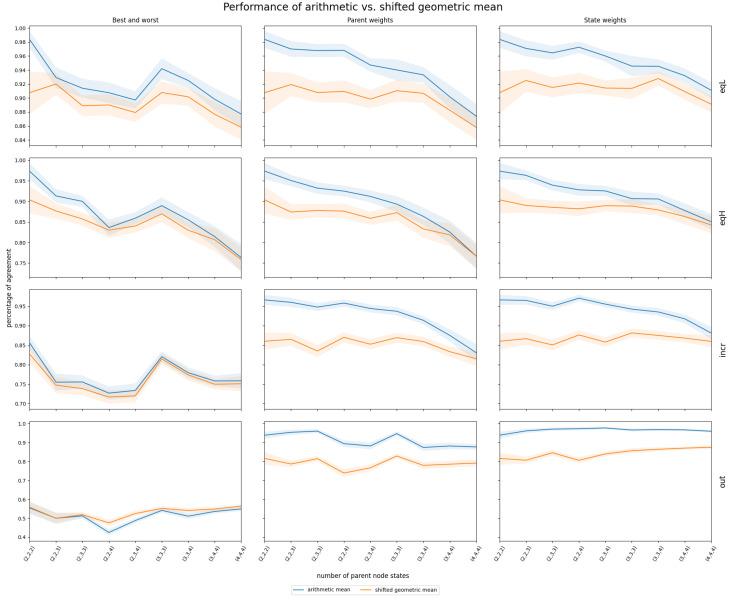
Mean and 95% confidence interval of InterBeta performance (percentage of agreement) over 100 replications. Using simulated data with four different correlation structures, the arithmetic and shifted geometric means are compared, with α and β as interpolation parameters.

Simulated data based on the correlation structures described in Section 2.1.2 provided insights into the performance of InterBeta with either the arithmetic or shifted geometric means. The geometric and harmonic means were not considered, as they assigned zero row weight to many CPT rows. The accuracy results measured by the KL divergence are shown in Figure 3. Figure A4 in Appendix A depicts the percentage of agreement results. The x-axis indicates the number of states for each parent node, ranging from two to four, represented as $\left(s_{1},s_{2},s_{3}\right)$, where $s_{i}$ is the number of states for parent *i*. Also, it should be noted that the row weights version of InterBeta is not included in this figure, as this version does not use a mean function. Finally, the α and β parameters of the beta distribution were interpolated.

The arithmetic mean consistently outperformed or matched the shifted geometric mean across various correlation structures and numbers of parent node states. This performance gap was particularly pronounced in the parent and state weights versions. For the correlation structures EqL, EqH, and Incr, the difference in performance diminished as the number of parent nodes increased. When parent nodes had three or more states, the 95% confidence intervals for both mean functions widened and overlapped, suggesting comparable performance. Future research could explore a broader range of parent node states to further investigate this trend. For the Out correlation structure, the performance difference between the arithmetic and shifted geometric means was minimal when using the best–worst rows version of InterBeta. However, when parent or state weights were included, the arithmetic mean exhibited a significantly lower mean KL divergence, indicating superior performance.

The percentage of agreement results, as depicted in Figure A4 in Appendix A, generally favored the arithmetic mean over the shifted geometric mean, except for the Out correlation structure when using the best–worst rows version of InterBeta.

In general, the arithmetic mean is recommended over the shifted geometric mean across most tested scenarios. The shifted geometric mean may only be preferable when the correlation structure includes an outlier and the best–worst rows version of InterBeta is used, as shown in Figure A4. However, since using (parent) weights is advised when outliers are known, situations in which the shifted geometric mean outperforms the geometric mean should rarely occur. Based on the simulation results, the arithmetic mean is the preferred choice, especially when parent nodes have up to four states.

#### 3.2.2. ExtraBeta

ExtraBeta, the extension of InterBeta that does not fix which CPT rows need to be elicited, was not included in the comparison of all CPT construction methods, as there would be too many results to compare. ExtraBeta was applied to reconstruct both the elicited CPTs and a collection of simulated CPTs. The results are reported for each combination of a good and a bad row as input for ExtraBeta. In addition, dominating parents were identified. A dominating parent is a parent node that has a significantly larger influence on the child node distribution than the other parent nodes and therefore is given a larger weight.

**Figure 4:**
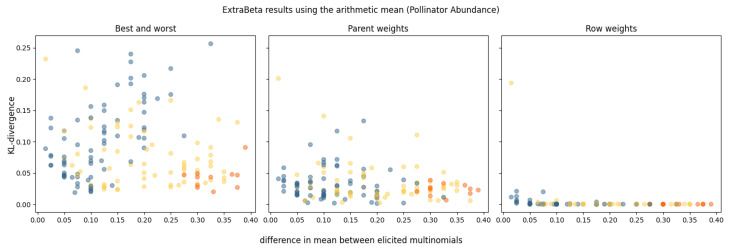
Results of reconstructing the pollinator abundance CPTs using ExtraBeta (arithmetic, α/β), based on all possible combinations of “good” and “bad” rows as input. The KL divergence is plotted against the absolute difference between the means of the input good and bad rows. The figure includes the InterBeta results (orange), the results where either the best row or the worst row was included as input (yellow), and the results where both the best and worst rows were not used (blue).

For the pollinator abundance CPT, no dominating parent was identified in any of the experts’ CPTs. Sixteen potential combinations of good and bad rows were tested for each expert’s assessments, and the results are shown in Figure 4. The performance, measured in terms of the mean KL divergence, is presented against the absolute difference between the means of the input good and bad rows. The orange dots represent the results of InterBeta, which is equivalent to having the best and worst rows as input for ExtraBeta. The yellow dots are the results when either the best row or the worst row was included in the input, and the blue dots represent the results when the best and worst rows were not used.

A noticeable trend emerged, indicating that as the difference between the means of the good row and bad row increased, the accuracy of ExtraBeta improved. This trend was most evident in the parent weights version, with a less pronounced yet observable effect in the best–worst rows version. Across all versions, numerous combinations of input rows—excluding the best or worst rows—performed as well as, or even better than, the standard InterBeta method. In the row weights version, performance differences between the various input rows were minimal, particularly when the mean difference exceeded 0.2, where no significant performance disparities were observed.

**Figure 5:**
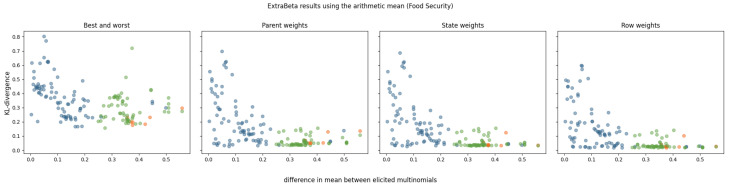
KL divergence results for reconstructing food security CPTs using ExtraBeta (arithmetic, α/β), based on possible combinations of “good” and “bad” rows as input. The figure includes the InterBeta results (orange), the results where the good row had *equivalized income* in its best state and the bad row had *equivalized income* in its worst state (green), and the results for the remaining combinations (blue).

For each of the food security CPTs, there were 25 combinations of good and bad rows. The results of using ExtraBeta are shown in Figure 5. It was found that the equivalized income had the largest influence on the child node [28]. In this figure, the dots are colored green for when the equivalized income was in its best state for the good row and in its worst state for the bad row.

The downward trend was even more evident than it was in the pollinator abundance CPTs. For the parent weights, state weights, and row weights versions, an elbow is visible in the graph when the difference between the means of the elicited multinomials was around 0.25. If the dominating parent was fixed to the extreme states as input for ExtraBeta, the performance was generally better than when using other input rows. Especially for the parent, state, and row weights versions of ExtraBeta, the performance was close to that of InterBeta when fixing the dominant parent node state. Fixing the dominant parent to its extreme states enforced the input rows to be in significantly different scenarios, resulting in relatively large differences between the input row means.

**Figure 6:**
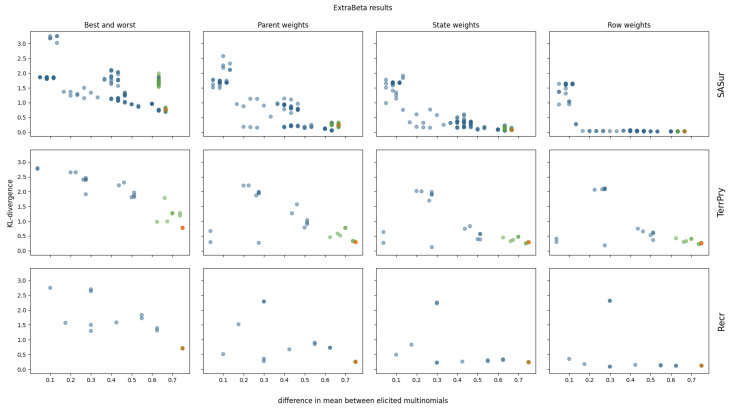
KL divergence results for reconstructing three polar bear CPTs using ExtraBeta (arithmetic, α/β), based on all possible combinations of “good” and “bad” rows as input. The figure includes the InterBeta results (orange); the results of the dominant parent node fixed to its best and worst states for the good and bad rows, respectively (green); and the results for the remaining combinations (blue).

**Figure A5:**
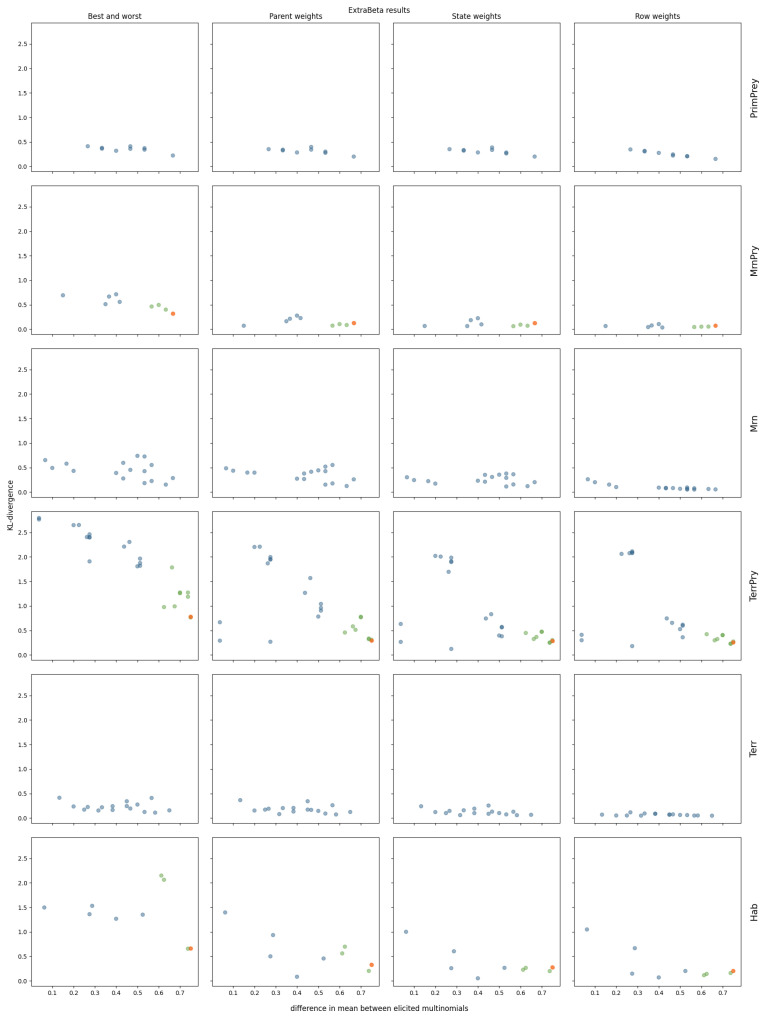
Results of reconstructing the polar bear CPTs using ExtraBeta (arithmetic, α/β), based on all possible combinations of good and bad rows as input. The figure includes the InterBeta results (orange); the results with the dominant parent node fixed to its best and worst states for the good and bad rows, respectively (green); and the results for the remaining combinations (blue).

**Figure A6:**
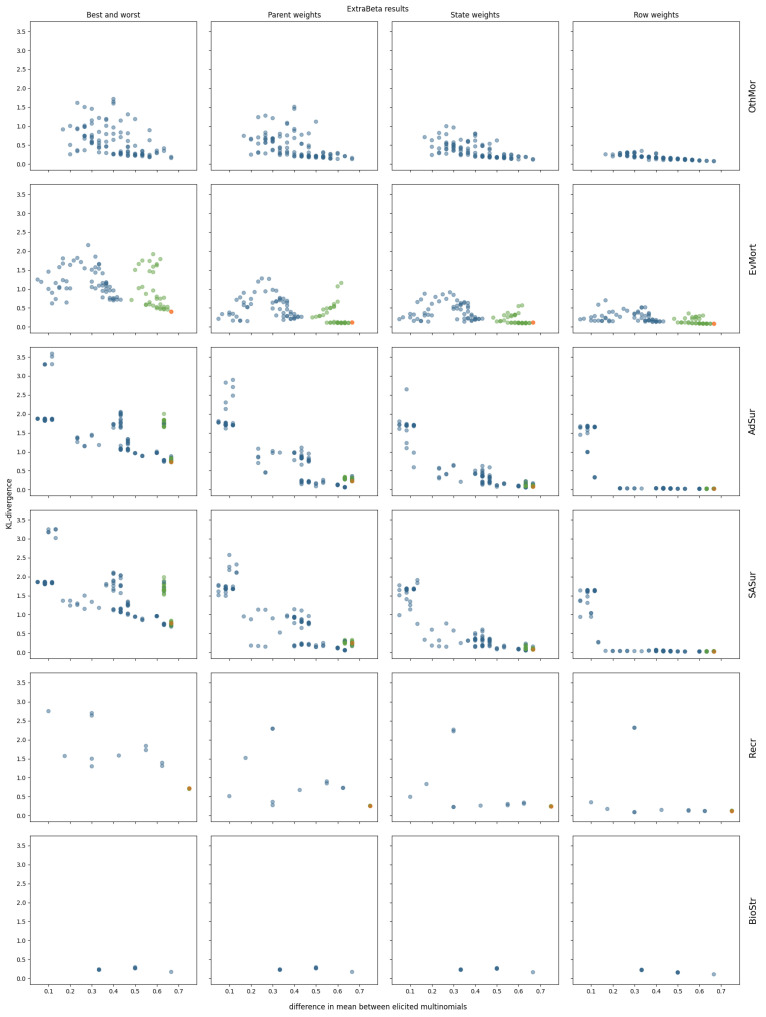
Results of reconstructing the polar bear CPTs using ExtraBeta (arithmetic, α/β), based on all possible combinations of good and bad rows as input. The figure includes the InterBeta results (orange); the results with the dominant parent node fixed to its best and worst states for the good and bad rows, respectively (green); and the results for the remaining combinations (blue).

The results of reconstructing all considered CPTs of the polar bear BN can be found in Figure A5 and Figure A6 in Appendix A. Four of the polar bear CPTs were not considered, as they were too large to test all possible input row combinations within a reasonable time. Note that these CPTs each had more than 144 values. The ExtraBeta reconstruction results of three selected CPTs are shown in Figure 6.

As observed for the pollinator abundance and food security CPTs, there was a negative correlation between the mean KL divergence and the difference between the input row means. This suggests that adding more weights to the method further reduced the mean KL divergence across all input row combinations. Specifically, in the case of ExtraBeta applied to the SASur CPT, the performance plateaued when the mean difference of the input rows exceeded 0.5 for the parent and state weights versions, and this plateau occurred at a mean difference of 0.2 for the row weights versions.

Fixing the dominating parent node once again ensured a large mean difference between the input rows. Consequently, the ExtraBeta results closely aligned with the InterBeta outcomes. In the Recr CPT, the similarity was particularly pronounced due to the parent node’s full dominance. When this parent was in its best state, the child node distribution mirrored the best row, and when it was in its worst state, it mirrored the worst row. As weights were introduced into both ExtraBeta and InterBeta, the performance gap between the two methods significantly reduced.

In addition to the elicited CPTs, ExtraBeta was also applied to reconstruct the simulated CPTs. Along with the correlation structure introduced in Section 2.1.2, two additional correlation structures that contained an outlier were considered. In this context, an outlier corresponds to a dominating parent. The two new correlation structures were variations of the Out correlation structure but with less pronounced outliers. The following two Spearman’s rank correlation matrices were used:

$$\begin{array}{cc} \mathrm{OutL}:\; & \;\left[\begin{array}{cccc} 1 & 0 & 0 & 0.25\\ 0 & 1 & 0 & 0.25\\ 0 & 0 & 1 & 0.5\\ 0.25 & 0.25 & 0.5 & 1 \end{array}\right]\;\;\mathrm{OutM}:\;\;\left[\begin{array}{cccc} 1 & 0 & 0 & 0.2\\ 0 & 1 & 0 & 0.2\\ 0 & 0 & 1 & 0.7\\ 0.2 & 0.2 & 0.7 & 1 \end{array}\right]. \end{array}$$

The CPT simulation methodology is detailed in [28]. One hundred simulations were performed, and for each replication, a discrete BN was fitted to the simulated data. Note that the structure remained the same throughout all the simulations (one child node with three parents). Similar to the CPTs from the expert judgment studies, the researched methods were tested for the CPTs obtained from the simulated data.

**Figure 7:**
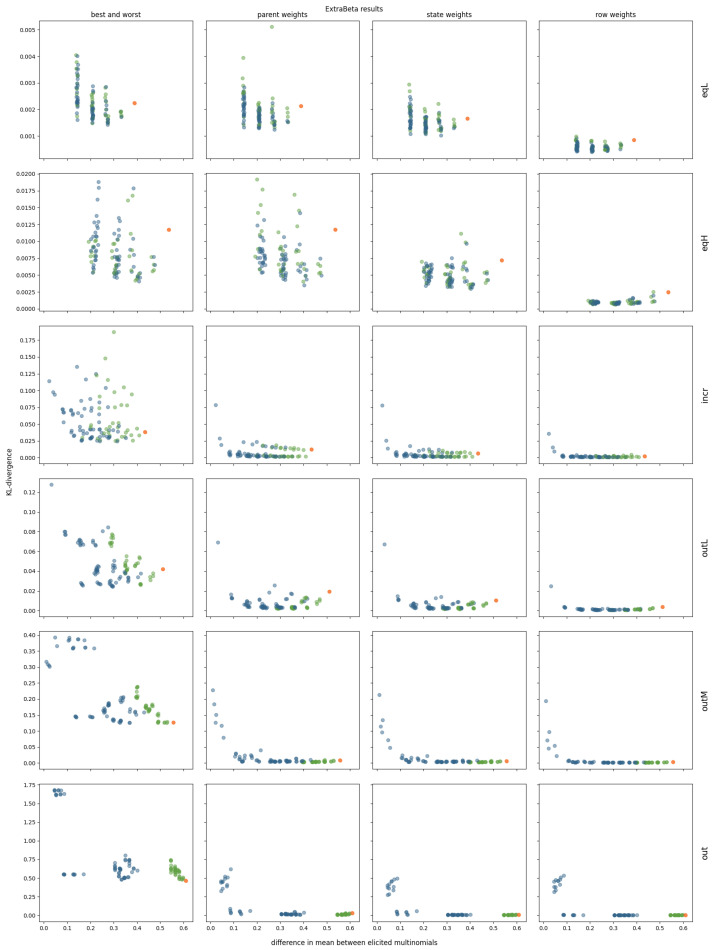
Mean KL divergence results of reconstructing 10 repetitions of simulated CPTs using ExtraBeta (arithmetic, α/β), with all potential combinations of “good” and “bad” rows as input. Includes the InterBeta results (orange), the results where the good row had the dominating parent in its best state and the bad row had the dominating parent in its worst state (green), and the results for the remaining combinations (blue).

The results are shown in Figure 7. For the EqL and EqH correlation structures, setting the third parent node to its extreme states had minimal impact, as this parent was not really a dominating parent node. Notably, InterBeta’s performance was relatively poor, with ExtraBeta demonstrating superior reconstruction accuracy in over half of the tested input row combinations. Once again, the accuracy improved as the difference in the means of the input rows increased.

For the Incr correlation structure, the effect of setting the third parent, with the largest parent–child correlation, to its extremes was significant. A downward trend in the KL divergence relative to the difference between the input multinomial means remained present, particularly in ExtraBeta versions incorporating parent, state, or row weights. For these versions, fixing the dominant parent to its extreme states resulted in CPTs with accuracy levels comparable to those generated by InterBeta.

For correlation structures with a clear outlier—OutL, OutM, and Out—the accuracy trend was even more pronounced, and incorporating weights as input significantly enhanced CPT reconstruction accuracy. In the OutL structure, ExtraBeta with fixed dominant parent states outperformed InterBeta when considering parent, state, or row weights. For OutM and Out, the CPT reconstruction accuracy of ExtraBeta with fixed dominant parent states was not significantly different from that of InterBeta in these weighted versions.

In general, for part of the good and bad input rows of ExtraBeta, the results were very similar to those of InterBeta. In some cases, the performance of ExtraBeta was better than that of InterBeta. However, the improved performance came at the cost of a slightly increased elicitation burden, since experts were asked to choose two rows. To increase the likelihood of experts selecting rows as input that perform similarly to, or better than, InterBeta, the difference between the means of the input rows should be as large as possible. In an elicitation, experts should therefore be guided in the row-selection process.

**Figure A7:**
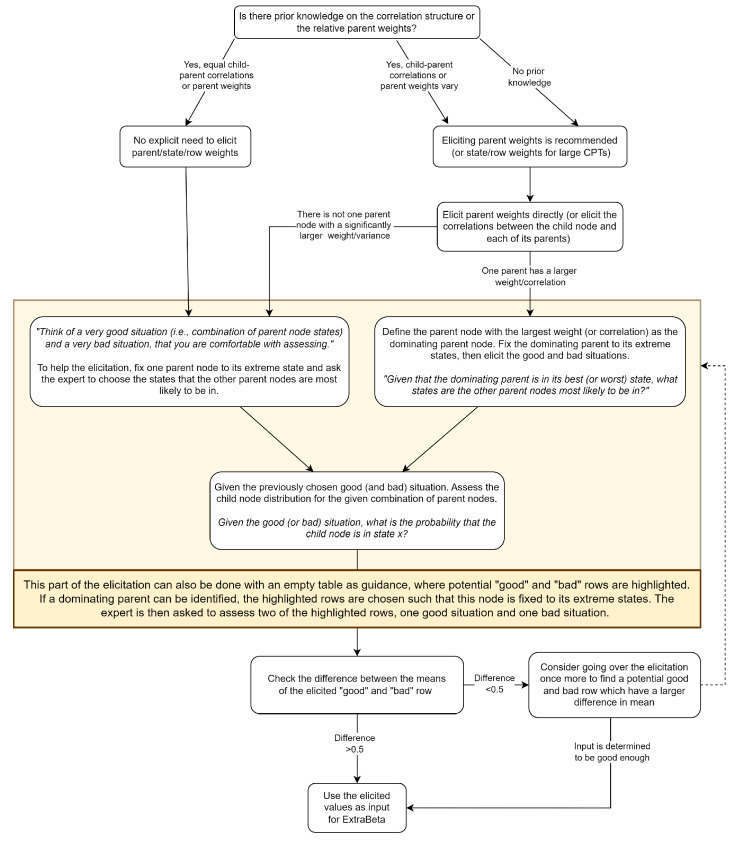
Proposed elicitation protocol for ExtraBeta.

Based on the simulation results, a potential elicitation protocol for ExtraBeta is proposed in Figure A7 in Appendix A. This protocol provides elicitation guidelines based on prior knowledge of parent influence or potential weights when such knowledge is absent.

In practice, if experts are familiar with CPTs, an empty CPT with highlighted good and bad rows can be shown for assessment. After eliciting two input rows, the modeler should review them—if their means are too similar, the process may be repeated. Otherwise, the rows and weights can be used in the ExtraBeta method.

## 4. Discussion

This study compared methods for constructing CPTs, focusing on the trade-off between the elicitation burden and accuracy. RNM, Functional Interpolation, and InterBeta were implemented in Python, with parameter optimization using grid and greedy search algorithms. While these algorithms achieved high accuracy, their real-world application depends on expert input, introducing uncertainty in parameter precision. This study assumed that experts can estimate (parent/state) weights to the nearest 0.5 and row weights to two decimal places, highlighting a key consideration regarding method robustness.

In practice, fatigue from fully eliciting CPTs could result in less thorough assessments. This highlights a potential advantage of CPT construction methods, such as InterBeta, which require fewer parameters and may yield more consistent results. Although these methods do not perfectly replicate fully elicited CPTs, the CPTs they construct may still be closer to the “ground truth”, as they avoid the pitfalls of expert fatigue.

Parameter flexibility proved beneficial only for the row weights version, with limited gains for parent and state weights. While further testing on simulated CPTs may not be warranted, exploring alternative input rows—like those used in Functional Interpolation—may yield better results.

ExtraBeta, a variant of InterBeta, aims to increase input flexibility by allowing experts to assess different “good” and “bad” rows instead of the strict best and worst rows. This adaptation can help mitigate biases, particularly when experts are asked to assess unfamiliar scenarios. Allowing them to assess rows that are within their frame of knowledge could improve the accuracy of the constructed CPTs and reduce the effects of cognitive biases.

In the simulation study comparing the arithmetic and shifted geometric means for InterBeta, the arithmetic mean consistently outperformed the shifted geometric mean across different correlation structures. However, the study was limited to parent nodes with up to four states. As the number of states increased, performance differences narrowed, highlighting the need for future research on methods handling more than four states.

Finally, this study explored the impact of dominant parent nodes on ExtraBeta’s accuracy. When a dominating parent was present, and parent, state, or row weights were used, ExtraBeta’s performance matched that of InterBeta. Even in scenarios without a dominant parent, ExtraBeta performed comparably to InterBeta for many input rows. Future studies should further investigate the proposed elicitation guidelines and how optimized weights change when different input rows are used with ExtraBeta.

A key limitation of this research is the reduced number of studies considered. While simulations helped address this, they were not able to capture all aspects. More studies would provide greater expert diversity and more varied elicitation or aggregation methods. Ideally, diverse expert groups should be used to evaluate each method’s performance under controlled conditions.

### Recommendations

Two main recommendations for future research follow from the above discussion. First, the comparison of the elicitation burden could be improved by examining whether some parameter types are more burdensome to elicit than others. This would refine the burden/accuracy trade-off across CPT construction methods. Case studies with timed tasks, expert surveys, and calibration exercises could offer valuable insights into parameter-specific burden.

Second, a case study using InterBeta or ExtraBeta is a key next step for validation with real experts. Ideally, multiple expert groups would follow different elicitation protocols, including full CPTs, InterBeta variants, and ExtraBeta. If expert availability is limited, a student-based calibration exercise could serve as a practical alternative.

## 5. Conclusions

This study compared the accuracy and elicitation burden of InterBeta, the Ranked Nodes Method, and Functional Interpolation in reconstructing previously elicited CPTs. InterBeta emerged as the best-performing method, particularly the parent weights version, which balanced good accuracy with low burden. The best–worst rows version of InterBeta required fewer elicited parameters than the parent weights version but performed considerably worse in terms of accuracy.

Based on the results of the comparison between the arithmetic and shifted geometric means, it was found that, for the tested scenarios, the arithmetic mean performed best. However, the performance difference between the means diminished as the number of parent node states increased.

The newly proposed ExtraBeta shows promise as an extension to InterBeta, especially when a dominant parent node can be identified, making its performance comparable to that of InterBeta. ExtraBeta requires experts to select two rows for assessment, which may increase the elicitation burden but could allow experts to evaluate scenarios with which they are more comfortable. The potential gain in accuracy might justify this added effort, although the impact may vary by expert group, subject matter, and the likelihood of ever observing extreme (best and worst) cases. Further research is needed to draw definitive conclusions.

## Figures and Tables

**Table 1:** Overview of the number of elicited input parameters for each version of the InterBeta method and additional assumptions.

InterBeta Version	# Parameters	Additional Assumptions
Row weights	$2\cdot s_{c}+\prod_{i=1}^{n}s_{i}$	Beta distribution with linearly interpolated parameters between best and worst rows.
Parent state weights	$2\cdot s_{c}+\sum_{i=1}^{n}s_{i}$	No parental synergy (i.e., increased combined effects of parent nodes).
Parent weights	$2\cdot s_{c}+n$	Uniformly increasing influence of states for each parent.
Best–worst rows	$2\cdot s_{c}$	Equal weights for each parent node.
Default	0	Beta(4,1), Beta(1,4) to model best and worst rows.

**Table 2:** Comparison of the mean KL divergence and the percentage of agreement results with respect to the number of elicited parameters for PB Ice CPTs constructed using variants of the InterBeta, RNM, and Functional Interpolation methods.

		Mean KL Divergence	Percentage of Agreement	Number of Elicited Parameters
InterBeta	Best–worst rows	0.23	70.8%	8
	Parent weights	0.13	76.4%	11
	State weights	0.12	73.6%	18
	Row weights	0.03	81.9%	78
InterBeta with elicited middle rows	Best–worst rows	1.03	66.7%	12
	Parent weights	0.65	80.6%	15
	State weights	0.32	69.4%	22
	Row weights	0.06	83.3%	82
Functional Interpolation	Normal	0.30	61.1%	32
	Truncated normal	0.38	70.8%	32
	Alpha/beta	0.25	62.5%	32
RNM		0.35	56.9%	16
AutoRNM		0.28	54.2%	32

## Data Availability

The code for each of the methods, including the optimization algorithms and the generation of simulation data is available at https://github.com/Bodille1/BN_parameterization.

## Appendix A

**Table A1:** Results of the KL divergence and the percentage of agreement performance for InterBeta, rounded to four decimals and one decimal, respectively. This table contains the information shown in Figure A3.

CPT	RNM		Functional Interpolation		
**(KL div. / % Agreement)**	**Original**	**AutoRNM**	**Normal**	**t-Normal**	**Beta**
Polar bears Ice	0.3509/56.9	0.2833/54.2	0.2996/61.1	0.3769/70.8	0.2501/62.5
Polar bears Disturb	0.1205/60.5	0.1547/48.1	0.0913/80.2	0.1525/85.2	0.0912/80.2
Polar bears CumPop	0.2119/72.2	0.2324/72.2	0.2163/75.0	1.5414/55.6	0.2015/75.0
Polar bears AFBod	0.186/91.7	0.2327/97.2	0.3125/69.4	0.6553/50.0	0.1522/91.7
Polar bears SASur	0.3026/63.9	0.3204/72.2	0.4699/63.9	0.8802/61.1	0.1005/94.4
Polar bears AdSur	0.3113/63.9	0.3312/72.2	0.5344/63.9	0.8427/55.6	0.124/94.4
Polar bears OthMor	0.148/77.8	0.181/70.4	0.1712/74.1	0.2521/74.1	0.1061/85.2
Polar bears EvMort	0.1332/100.0	0.1518/100.0	0.1833/92.6	0.3768/66.7	0.1031/92.6
Polar bears TerrPry	0.22/100.0	0.2745/100.0	0.4597/62.5	0.9088/50.0	0.2109/100.0
Polar bears Recr	0.2418/100.0	0.2596/100.0	0.6192/75.0	0.7533/58.3	0.2718/91.7
Polar bears Mrn	0.138/50.0	0.1412/75.0	0.1911/75.0	0.4872/75.0	0.1008/91.7
Polar bears Hab	0.417/55.6	0.4197/55.6	0.178/77.8	0.9017/55.6	0.1474/77.8
Polar bears Terr	0.0923/91.7	0.0999/91.7	0.0846/91.7	0.6179/75.0	0.1288/91.7
Polar bears PrimPrey	0.1983/77.8	0.227/77.8	0.2511/100.0	0.2791/66.7	0.2115/77.8
Polar bears MrnPry	0.0862/88.9	0.1425/88.9	0.0594/100.0	0.5905/77.8	0.0688/88.9
Polar bears BioStr	0.1472/77.8	0.1633/77.8	0.2262/66.7	0.2582/66.7	0.1417/77.8
Food security EWDM	0.1843/66.7	0.1851/66.7	0.1043/91.7	0.0631/91.7	0.0171/91.7
Food security PWDM	0.1863/50.0	0.1752/58.3	0.1005/75.0	0.109/66.7	0.0242/66.7
Food security 1	0.188/66.7	0.1733/58.3	0.1221/75.0	0.1558/66.7	0.0237/66.7
Food security 2	0.2108/58.3	0.1848/41.7	0.1238/75.0	0.1073/66.7	0.0284/66.7
Food security 3	0.2005/50.0	0.1697/75.0	0.1299/66.7	0.1831/58.3	0.0816/58.3
Food security 4	0.1903/66.7	0.2223/58.3	0.0852/100.0	0.0758/75.0	0.0235/83.3
Food security 5	0.4594/41.7	0.4761/66.7	0.5775/66.7	0.1192/100.0	0.028/100.0
Pollinator abundance EWDM	0.0032/100.0	0.0031/100.0	0.0/100.0	0.0/100.0	0.0/100.0
Pollinator abundance 1	0.01/100.0	0.0099/100.0	0.0/100.0	0.0/100.0	0.0/100.0
Pollinator abundance 2	0.0094/100.0	0.0082/100.0	0.0/100.0	0.0/87.5	0.0/100.0
Pollinator abundance 3	0.0088/100.0	0.0083/100.0	0.0/100.0	0.0/87.5	0.0/100.0
Pollinator abundance 4	0.004/100.0	0.0027/100.0	0.0/100.0	0.0/100.0	0.0/100.0
Pollinator abundance 5	0.0201/87.5	0.0161/100.0	0.0/100.0	0.0/100.0	0.0/100.0
Pollinator abundance 6	0.0043/100.0	0.0054/100.0	0.0/100.0	0.0/100.0	0.0/100.0
Pollinator abundance 7	0.0166/75.0	0.0153/87.5	0.0/100.0	0.0/100.0	0.0/100.0
Pollinator abundance 8	0.0247/75.0	0.0238/75.0	0.0/100.0	0.0/87.5	0.0/100.0
Pollinator abundance 9	0.0219/87.5	0.0201/75.0	0.0/100.0	0.0/100.0	0.0/100.0
Pollinator abundance 10	0.0086/100.0	0.0071/100.0	0.0/100.0	0.0/100.0	0.0/100.0

**Table A2:** Results of the KL divergence and the percentage of agreement performance for InterBeta, rounded to four decimals and one decimal, respectively. This table contains the information shown in Figure A3.

CPT	InterBeta			
**(KL div. / % Agreement)**	**Best–Worst Rows**	**Parent Weights**	**State Weights**	**Row Weights**
Polar bears Ice	0.2284/70.8	0.1347/76.4	0.1158/73.6	0.0337/81.9
Polar bears Disturb	0.1885/75.3	0.1885/75.3	0.1853/72.8	0.1346/67.9
Polar bears CumPop	0.3701/63.9	0.3554/72.2	0.3407/77.8	0.2367/88.9
Polar bears AFBod	0.704/47.2	0.2047/97.2	0.1799/97.2	0.1324/97.2
Polar bears SASur	0.6735/58.3	0.2423/75.0	0.0792/97.2	0.0298/100.0
Polar bears AdSur	0.6897/58.3	0.201/80.6	0.0753/97.2	0.0256/100.0
Polar bears OthMor	0.1616/77.8	0.133/77.8	0.1188/77.8	0.0766/85.2
Polar bears EvMort	0.4037/55.6	0.1105/100.0	0.1096/100.0	0.084/92.6
Polar bears TerrPry	0.7762/62.5	0.2977/100.0	0.2896/100.0	0.258/100.0
Polar bears Recr	0.7226/58.3	0.2264/100.0	0.149/100.0	0.1165/91.7
Polar bears Mrn	0.2361/83.3	0.2015/83.3	0.1302/100.0	0.0545/100.0
Polar bears Hab	0.6701/55.6	0.3285/66.7	0.2724/77.8	0.1915/88.9
Polar bears Terr	0.1646/91.7	0.1394/91.7	0.0612/83.3	0.0478/100.0
Polar bears PrimPrey	0.2239/66.7	0.2021/100.0	0.2019/100.0	0.1528/88.9
Polar bears MrnPry	0.341/66.7	0.1533/88.9	0.1445/88.9	0.0707/100.0
Polar bears BioStr	0.1641/77.8	0.1641/77.8	0.1602/88.9	0.1063/77.8
Food security EWDM	0.1819/66.7	0.0288/91.7	0.0285/91.7	0.0221/91.7
Food security PWDM	0.1987/41.7	0.0273/83.3	0.0272/83.3	0.0184/75.0
Food security 1	0.1756/50.0	0.0477/75.0	0.0381/75.0	0.0163/75.0
Food security 2	0.1982/50.0	0.0348/91.7	0.0348/91.7	0.0247/75.0
Food security 3	0.2111/66.7	0.1114/91.7	0.1054/91.7	0.0638/83.3
Food security 4	0.2164/50.0	0.0346/75.0	0.0342/75.0	0.0214/83.3
Food security 5	0.2759/75.0	0.11/100.0	0.0307/100.0	0.0233/100.0
Pollinator abundance EWDM	0.0164/87.5	0.0027/100.0	0.0027/100.0	0.0/100.0
Pollinator abundance 1	0.0265/87.5	0.0133/100.0	0.0133/100.0	0.0/100.0
Pollinator abundance 2	0.0374/87.5	0.0063/100.0	0.0063/100.0	0.0/100.0
Pollinator abundance 3	0.0132/87.5	0.0097/87.5	0.0097/87.5	0.0/100.0
Pollinator abundance 4	0.041/87.5	0.0031/100.0	0.0031/100.0	0.0/100.0
Pollinator abundance 5	0.0391/100.0	0.024/100.0	0.024/100.0	0.0/100.0
Pollinator abundance 6	0.0405/87.5	0.0141/100.0	0.0141/100.0	0.0/100.0
Pollinator abundance 7	0.0235/100.0	0.012/100.0	0.012/100.0	0.0/100.0
Pollinator abundance 8	0.042/87.5	0.0218/87.5	0.0218/87.5	0.0/100.0
Pollinator abundance 9	0.0318/87.5	0.0206/87.5	0.0206/87.5	0.0/100.0
Pollinator abundance 10	0.0553/87.5	0.0046/100.0	0.0046/100.0	0.0/100.0

**Table A3:** Results of the KL divergence and the percentage of agreement performance for InterBeta when the middle rows are also elicited, rounded to four decimals and one decimal, respectively. This table contains the information shown in Figure A3.

CPT	InterBeta with Elicited Middle Rows			
**(KL div. / % Agreement)**	**Best, Worst, Mid**	**Parent Weights**	**State Weights**	**Row Weights**
Polar bears Ice	1.0349/66.7	0.6539/80.6	0.3214/69.4	0.0636/83.3
Polar bears Disturb	1.2857/75.3	1.2857/75.3	1.27/75.3	0.6246/82.7
Polar bears CumPop	0.7845/66.7	0.4828/63.9	0.4663/66.7	0.1197/86.1
Polar bears AFBod	1.7942/47.2	0.3596/97.2	0.1425/88.9	0.0584/91.7
Polar bears SASur	1.6724/58.3	0.2478/88.9	0.223/97.2	0.0337/100.0
Polar bears AdSur	1.6483/58.3	0.299/88.9	0.2623/97.2	0.0277/100.0
Polar bears OthMor	0.8206/77.8	0.7762/77.8	0.7429/77.8	0.2117/92.6
Polar bears EvMort	1.312/55.6	0.3012/100.0	0.3003/100.0	0.1189/96.3
Polar bears TerrPry	1.6725/56.2	0.0618/100.0	0.0524/100.0	0.0441/100.0
Polar bears Recr	1.702/50.0	0.4709/100.0	0.1893/100.0	0.0501/100.0
Polar bears Mrn	0.6355/75.0	0.5673/91.7	0.3914/91.7	0.0722/100.0
Polar bears Hab	1.3242/55.6	0.075/88.9	0.0667/88.9	0.0584/100.0
Polar bears Terr	0.496/91.7	0.3498/91.7	0.3084/100.0	0.1547/100.0
Polar bears PrimPrey	1.0344/66.7	0.6767/100.0	0.6757/100.0	0.3311/77.8
Polar bears MrnPry	1.0919/66.7	0.2235/88.9	0.2043/100.0	0.0347/100.0
Polar bears BioStr	0.8446/77.8	0.7387/77.8	0.7387/77.8	0.3464/77.8
